# ERα36 Promotes *MDR1*-Mediated Adriamycin Resistance via Non-Genomic Signaling in Triple-Negative Breast Cancer

**DOI:** 10.3390/ijms26157200

**Published:** 2025-07-25

**Authors:** Muslimbek Mukhammad Ugli Poyonov, Anh Thi Ngoc Bui, Seung-Yeon Lee, Gi-Ho Lee, Hye-Gwang Jeong

**Affiliations:** College of Pharmacy, Chungnam National University, Daejeon 34134, Republic of Korea; poyonov@o.cnu.ac.kr (M.M.U.P.); anhbui@cnu.ac.kr (A.T.N.B.); sy9842@o.cnu.ac.kr (S.-Y.L.); ghk1900@cnu.ac.kr (G.-H.L.)

**Keywords:** estrogen receptor 36, P-glycoprotein, drug resistance, breast cancer, TNBC

## Abstract

Drug resistance remains a critical barrier to effective treatment in several cancers, particularly triple-negative breast cancer (TNBC). Estrogen receptor α36 (ERα36), a variant of the estrogen receptor in ER-negative breast cancer cells, plays important roles in cancer cell proliferation. We investigated the role of ERα36 in regulating multidrug resistance protein 1 (*MDR1*) in MDA-MB-231 human breast cancer cells. The activation of ERα36 by BSA-conjugated estradiol (BSA-E2) increased cell viability under Adriamycin exposure, suggesting its involvement in promoting drug resistance. BSA-E2 treatment significantly reduced the intracellular rhodamine-123 levels by activating the *MDR1* efflux function, which was linked to increased *MDR1* transcription and protein expression. The mechanical ERα36-mediated BSA-E2-induced activation of EGFR and downstream signaling via c-Src led to an activation of the Akt/ERK pathways and transcription factors, NF-κB and CREB. Additionally, ERα36 is involved in activating Wnt/β-catenin pathways to induce *MDR1* expression. The silencing of ERα36 inhibited the BSA-E2-induced phosphorylation of Akt and ERK, thereby reducing *MDR1* expression via downregulation of NF-κB and CREB as well as Wnt/β-catenin signaling. These findings demonstrated that ERα36 promotes *MDR1* expression through multiple non-genomic signaling cascades, including Akt/ERK-NF-κB/CREB and Wnt/β-catenin pathways, and highlight the role of ERα36 as a promising target to enhance chemotherapeutic efficacy in TNBC.

## 1. Introduction

Breast cancer presents as a global disease burden, ranking as the most prevalent cancer in women and the second most frequently diagnosed cancer overall [1]. Despite significant advancements in early detection and therapeutic strategies, treatment resistance remains a critical challenge in clinical oncology. Among various breast cancer subtypes, triple-negative breast cancer (TNBC) accounts for approximately 15–20% of all breast cancer cases and is mainly observed in younger women [2]. TNBC is defined by the lack of estrogen receptors (particularly estrogen receptor 66 alpha, ERα66), progesterone receptors (PRs), and human epidermal growth factor receptor 2 (HER2) expression in tumor cells. This absence of conventional therapeutic targets classified TNBC as receptor-negative, contributing to its poor prognosis and limited treatment options [3,4]. In clinical practice, chemotherapy remains the mainstay of systemic treatment for TNBC because it does not respond to endocrine therapies or HER2-targeted agents. Unfortunately, TNBC often develops multidrug resistance (MDR) and shows poor long-term outcomes [5].

The majority of breast tissue is hormone-dependent, especially on estradiol, and is controlled by estrogen secretion. Given this dependency, estrogen signaling is critically involved in the development and metastasis of breast cancer through genomic and non-genomic signaling pathways [6,7]. This signaling is mediated by estrogen receptors (ERs), primarily Erα and Erβ. These receptors are ligand-activated transcription factors that, upon binding to 17β-estradiol (E2)—the predominant estrogen in the body—form homo- or heterodimers (E2-ER complexes). These complexes then translocate to the nucleus to initiate the transcription of target genes via the genomic pathway [8]. In parallel, a rapid non-genomic pathway can be triggered by E2 binding to ERs located at the membrane or in the cytoplasm. Upon binding, it triggers kinase cascade activation and intracellular calcium signaling such as phosphatidylinositide3-kinase/protein kinase B (PI3K/Akt), mitogen-activated protein kinase/extracellular regulated kinase (MAPK/ERK), and cAMP/PKA pathways [9]. These activations influence multiple cellular processes like proliferation, migration, and survival [9,10,11].

In recent years, research attention has turned to alternative isoforms of ERs as potential targets for treating TNBC, which lacks the classical ERα66. Among ER isoforms, ERα36 has gained attention due to its distinct localization and function. ERα36 is a 36 kDa splice variant of ERα66 that lacks both transactivation domains AF-1 and AF-2 and possesses distinct N- and C-termini [12,13]. Unlike ERα66, ERα36 is primarily found at the plasma membrane and in the cytoplasm, where it mediates a rapid membrane-initiated non-genomic estrogen signaling pathway [14,15]. Through this pathway, ERα36 is linked to the imitation of MAPK and PI3K/Akt signaling cascades. Previous studies have demonstrated that MAPK and Akt activation are correlated with cell growth and proliferation as well as the invasion and metastasis of various cancers [16,17,18,19]. Notably, ERα36 is frequently co-expressed with membrane proteins, such as the epidermal growth factor receptor (EGFR), in TNBC cells. This co-expression contributes to tumor growth through the formation of proto-oncogene tyrosine protein kinase (Src)/EGFR signaling complexes [20,21,22]. Elevated ERα36 expression is particularly observed in ER-negative, TNBC, and HER2-positive breast cancers [20,23,24]. In TNBC tumors, ERα36 is correlated with drug resistance, suboptimal responses to chemotherapy, and the promotion of epithelial–mesenchymal transition (EMT). Clinical data indicate that patients whose tumors lack ERα36 expression respond more favorably to chemotherapy than those with ERα36-positive tumors [25]. Chaudhri et al. demonstrated that ERα36 enhances the proliferation of breast cancer cells by regulating the expression of adhesion molecules and proteins involved in EMT [26].

Multidrug resistance plays a critical role in limiting the efficacy of breast cancer treatment and contributes substantially to therapeutic failure. A key mechanism underlying MDR involves ATP-binding cassette (ABC) family transporters, also known as multidrug resistance transporters, which actively mediate drug efflux in cancer cells [27]. The ATP-dependent pumps enable cancer cells to evade cytotoxic effects by limiting intracellular drug accumulation. Alternative model suggests that P-glycoprotein (P-gp), encoded by the *MDR1* gene, may function as a flippase, transporting substrates from the cytosolic side to the external leaflet of the lipid bilayer [28,29]. Elevated *MDR1* (P-gp) expression promotes the active efflux of various hydrophobic anticancer drugs, ultimately reducing the efficacy of chemotherapy [30]. The transcription of *MDR1* is tightly regulated by various transcription factors. Among these, nuclear factor-κB (NF-κB), cAMP response elements (CREB), and β-catenin have been shown to bind to regulatory elements within the *MDR1* promoter and enhance its transcription [31]. These factors are known to be activated by membrane-initiated signaling pathways. For example, the activation of NF-κB through pathways involving Src kinase, PI3K/Akt, or MAPKs leads to IκB phosphorylation and degradation, which enables NF-κB nuclear translocation and the subsequent activation of genes involved in inflammation, survival, and drug resistance [32,33]. *MDR1* activation was reported to occur through NF-κB activation, linking inflammatory and survival signaling to drug resistance in cancer cells [34,35]. Emerging evidence indicates that ER signaling and the Wnt/β-catenin pathways converge to enhance *MDR1* expression. Both ERα and β-catenin can directly activate *MDR1* transcription via binding to its promoter region. In MCF7 breast cancer cells, the interaction of ERα with WW domain-binding protein 2 (WBP2) actively modulates *MDR1* expression and contributes to doxorubicin resistance [36]. Meanwhile, the Wnt/β-catenin signal is triggered in doxorubicin-induced MDR cancer cells, where β-catenin associates with the CREB binding protein (CBP) to drive *MDR1* expression in an MEK/ERK-dependent manner [37]. While biologically relevant crosstalk between ER and Wnt/β-catenin signaling has been implicated in fostering chemoresistance, the direct molecular synergy between these pathways in the regulation of *MDR1* has not been fully evaluated.

Although ERα36 is important for the tumorigenesis and progression in breast cancer, the specific mechanisms through which it contributes to *MDR1* expression in TNBC remain poorly understood. Addressing this gap, we aimed to elucidate the role of ERα36 in regulating *MDR1* expression in MDA-MB-231 breast cancer cells, with a particular focus on its potential crosstalk with the Wnt/β-catenin pathway. Delineating this relationship may provide insights into overcoming drug resistance in aggressive cancer subtypes.

## 2. Results

### 2.1. BSA-E2 Modulates Adriamycin Resistance by Enhancing MDR1-Mediated Efflux

**Figure 1:**
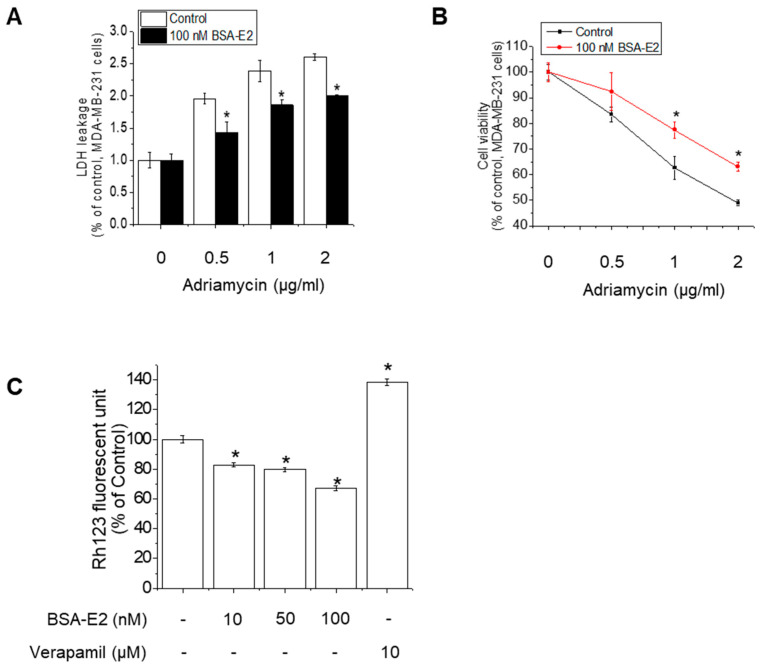
BSA-E2 modulates Adriamycin resistance by enhancing *MDR1*-mediated efflux. (**A**) The effect of BSA-E2 on Adriamycin-induced cytotoxicity. Cells were pretreated with 100 nM of BSA-E2 for 24 h, followed by incubation with various concentrations of Adriamycin (0.5–2 µg/mL) for 48 h. Cell cytotoxicity was determined by LDH assay. (**B**) Cell viability was measured by the MTT assay. (**C**) Effect of BSA-E2 on intracellular Rh-123 accumulation. Cells were pretreated with BSA-E2 (0–100 nM) or 10 µM of verapamil for 48 h and then exposed to 5 µM of Rh-123 for 90 min. The intracellular Rh-123 accumulation was quantified based on fluorescence intensity. Data shown are the means ± SD from three independent experiments. * significantly different from the control at *p* < 0.01.

Adriamycin (also known as doxorubicin) is a widely used chemotherapeutic agent, especially for treating breast cancer, leukemia, lymphomas, and sarcomas. However, Adriamycin resistance poses a major obstacle to effective cancer therapy. In ER-positive tumors, estrogen signaling is known to influence drug response. To specifically assess the involvement of membrane estrogen receptor signaling in Adriamycin-induced cytotoxicity, we employed BSA-conjugated estradiol (BSA-E2), which is membrane-impermeable and thus selectively activates membrane-associated estrogen receptors without triggering the genomic signaling pathway. By assessing cell viability following co-treatment with Adriamycin and BSA-E2, we realized that BSA-E2 increased the cell viability of MDA-MB-231 cells while it attenuated the cytotoxicity caused by Adriamycin (Figure 1A,B). Because Adriamycin-induced drug resistance is often associated with increased *MDR1* expression, we further investigated whether treatment with BSA-E2 suppresses the expression of *MDR1* using Rhodamine-123 (Rh-123) accumulation assays. Figure 1C shows that treatment with verapamil, an *MDR1* inhibitor, efficiently increased the accumulation of the Rh123 fluorescent signal, indicating that *MDR1* is associated with drug resistance. Cells treated with BSA-E2 showed a notable reduction in intracellular Rh-123 levels, suggesting that BSA-E2 likely increases *MDR1* activity or expression, thereby promoting drug efflux and potentially contributing to drug resistance.

### 2.2. BSA-E2 Increases MDR1 Expression in MDA-MB-231 Cells

**Figure 2:**
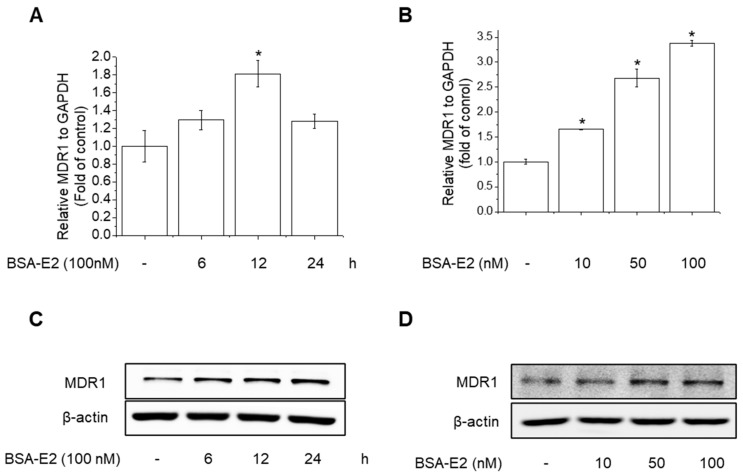
BSA-E2 induces *MDR1* expression in MDA-MB-231 cells. Cells were treated with 100 nM of BSA-E2 for 6–24 h (**A**) or with BSA-E2 (10–100 nM) for 12 h (**B**). mRNA levels were measured by qRT-PCR. *MDR1* protein expression was determined by Western blot. (**C**) Cells were treated with 100 nM of BSA-E2 for 24 h. (**D**) Cells were treated with BSA-E2 (10–100 nM) for 24 h. Data shown are the means ± SD from three independent experiments. * significantly different from the control at *p* < 0.01.

Since the development of the *MDR1* phenotype in cancer cells is linked to *MDR1* overexpression, we further examined the change in *MDR1* mRNA levels upon treatment with BSA-E2 using RT-PCR. BSA-E2 treatment significantly increased the mRNA level of *MDR1* in a time- and concentration-dependent manner (Figure 2A,B). Together, Western blot exhibited an elevated protein level of *MDR1* (Figure 2C,D; quantified in Supplementary Figure S1A,B). Taken together, these data suggest that BSA-E2 promotes *MDR1* expression in MDA-MB-231 cells.

### 2.3. BSA-E2 Induces MDR1 Expression via Activation of NF-κB and CREB Phosphorylation

**Figure 3:**
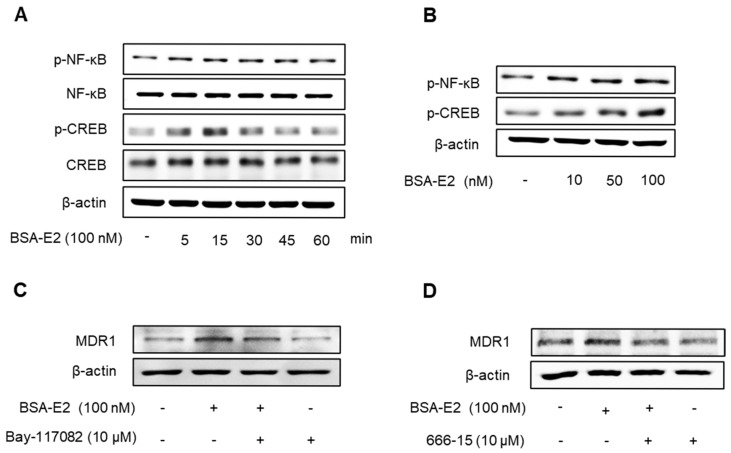
BSA-E2 enhances the *MDR1* level via the activation of NF-κB and CREB phosphorylation. Cells were treated with 100 nM of BSA-E2 for 5–60 min (**A**) or with BSA-E2 (10–100 nM) for 15 min (**B**). p-NF-κB and p-CREB protein levels were determined by Western blot. (**C**) The *MDR1* protein level was determined by Western blot in cells pretreated with the NF-κB inhibitor, Bay-117082 (10 µM) for 1 h, followed by 100 nM of BSA-E2 for 24 h. (**D**) The *MDR1* protein level was determined by Western blot in cells pretreated with the CREB inhibitor, 666-15 (10 µM) for 1 h, followed by 100 nM of BSA-E2 for 24 h.

*MDR1* expression is governed by a variety of transcription factors, including NF-κB, particularly the p65 (RelA) subunit, and CREB, in which CREB binds to the cAMP response element to modulate the expression of *MDR1* in breast cancer cells [38,39]. To investigate whether CREB and NF-κB activation are involved in Erα36-mediated *MDR1* expression in MDA-MB-231 cells, we examined the activation of CREB and NF-κB in response to BSA-E2 treatment. BSA-E2 significantly increased CREB and NF-κB phosphorylation in a time- and concentration-dependent manner (Figure 3A,B; quantified in Supplementary Figure S2A,B). To further clarify the effects of BSA-E2 on NF-κB and CREB in BSA-E2-induced *MDR1* expression, specific inhibitors, Bay 11-7082 (an NF-κB inhibitor) and 666-15 (a CREB inhibitor), were used. Pretreatment with Bay-117082 and 666-15 suppressed BSA-E2-induced *MDR1* expression (Figure 3C,D; quantified in Supplementary Figure S2C,D). These results suggest that the transcriptional activity of NF-κB and CREB is important for BSA-E2-mediated regulation of *MDR1* in MDA-MB-231 cells.

### 2.4. Akt/ERK Signaling Pathway Is Important for ERα36-Mediated MDR1 Expression

**Figure 4:**
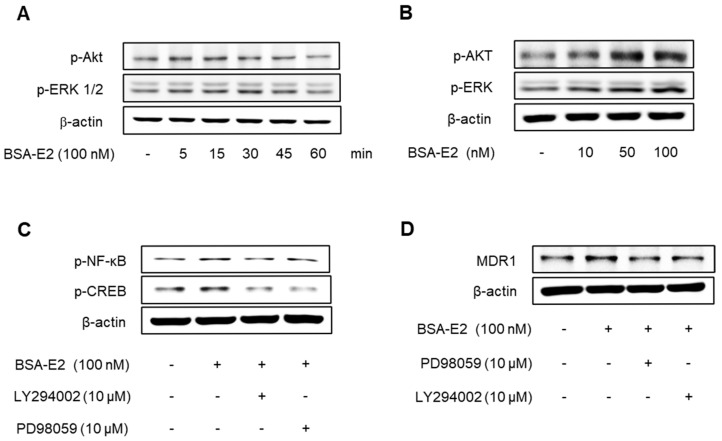
The Akt/ERK signaling pathway is important for the ERα36-mediated regulation of *MDR1* expression. Cells were treated with 100 nM of BSA-E2 for 5–60 min (**A**) or BSA-E2 (10−100 nM) for 15 min (**B**). p-Akt and p-ERK protein levels were determined by Western blot. (**C**) Cells were pretreated with 10 µM of LY294002 and 10 µM of PD98059 for 1 h, followed by treatment with 100 nM of BSA-E2 for 24 h. The *MDR1* protein level was determined by Western blot. (**D**) Cells were pretreated with 10 µM of LY294002 and 10 µM of PD98059 for 1 h and then treated with 100 nM of BSA-E2 for 15 min. CREB and NF-κB protein levels were analyzed by Western blot.

Given that NF-κB and CREB are regulated through the Akt and ERK signaling pathways, we next investigated whether BSA-E2 influences the activation of these upstream pathways. Cells treated with BSA-E2 exhibited an increase in the phosphorylation of Akt and ERK in a manner dependent on both time and concentration (Figure 4A,B; quantified in Supplementary Figure S3A,B). Interestingly, pretreatment of LY294002 (a PI3K/Akt inhibitor) and PD98059 (an ERK inhibitor) inhibited CREB and NF-κB phosphorylation as well as the expression of *MDR1* (Figure 4C,D; quantified in Supplementary Figure S3C,D). These findings indicate that Akt and ERK activity are crucial for *MDR1* upregulation by BSA-E2.

### 2.5. BSA-E2 Activates EGFR/Src-Akt/ERK Signaling to Induce MDR1 Expression

**Figure 5:**
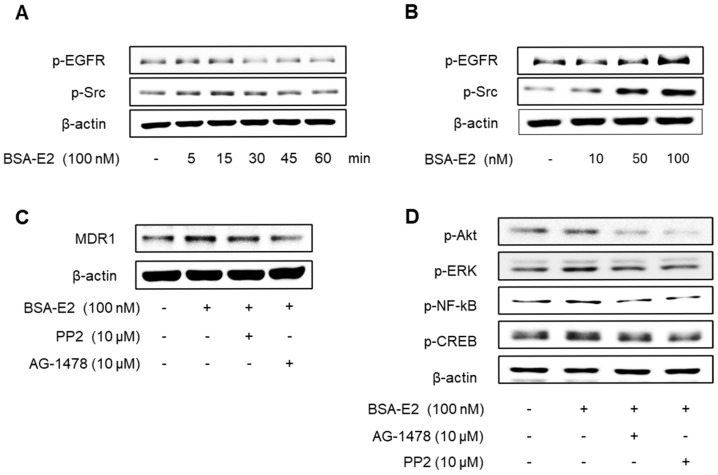
Effect of BSA-E2 on *MDR1* expression with the Src/EGFR signaling pathway. Cells were treated with 100 nM of BSA-E2 for 5−60 min (**A**) or BSA-E2 (10, 50, 100) for 5 min (**B**). p-Src and p-EGFR protein levels were determined by Western blot. (**C**) Cells were pretreated with 10 µM of PP2 and 10 µM of AG1478 for 1 h, followed by treatment with 100 nM of BSA-E2 for 24 h. *MDR1* protein levels were assessed by Western blot. (**D**) Cells were pretreated with 10 µM of AG1478 and 10 µM of PP2 for 1 h and then treated with 100 BSA-E2 for 30 min. Phosphorylation of Akt, ERK, NF-κB, and CREB was detected by Western blot.

Following the observed activation of Akt and ERK, we explored whether EGFR and Src—known upstream regulators of these pathways—are also activated in response to BSA-E2. As shown in Figure 5A,B, BSA-E2 induced the phosphorylation of EGFR and Src in MDA-MB-231 cells (the band density was quantified in Supplementary Figure S4A,B). Pretreatment with AG1478 (an EGFR inhibitor) or PP2 (a Src inhibitor) attenuated the BSA-E2-induced expression of *MDR1* and the phosphorylation of Akt, ERK, CREB, and NF-κB (Figure 5C,D; quantified in Supplementary Figure S5C,D), suggesting that the activation of EGFR and Src plays a key upstream role in regulating *MDR1* expression.

### 2.6. ERα36 Mediates BSA-E2-Induced Drug Resistance and Cell Survival in MDA-MB-231 Cells

**Figure 6:**
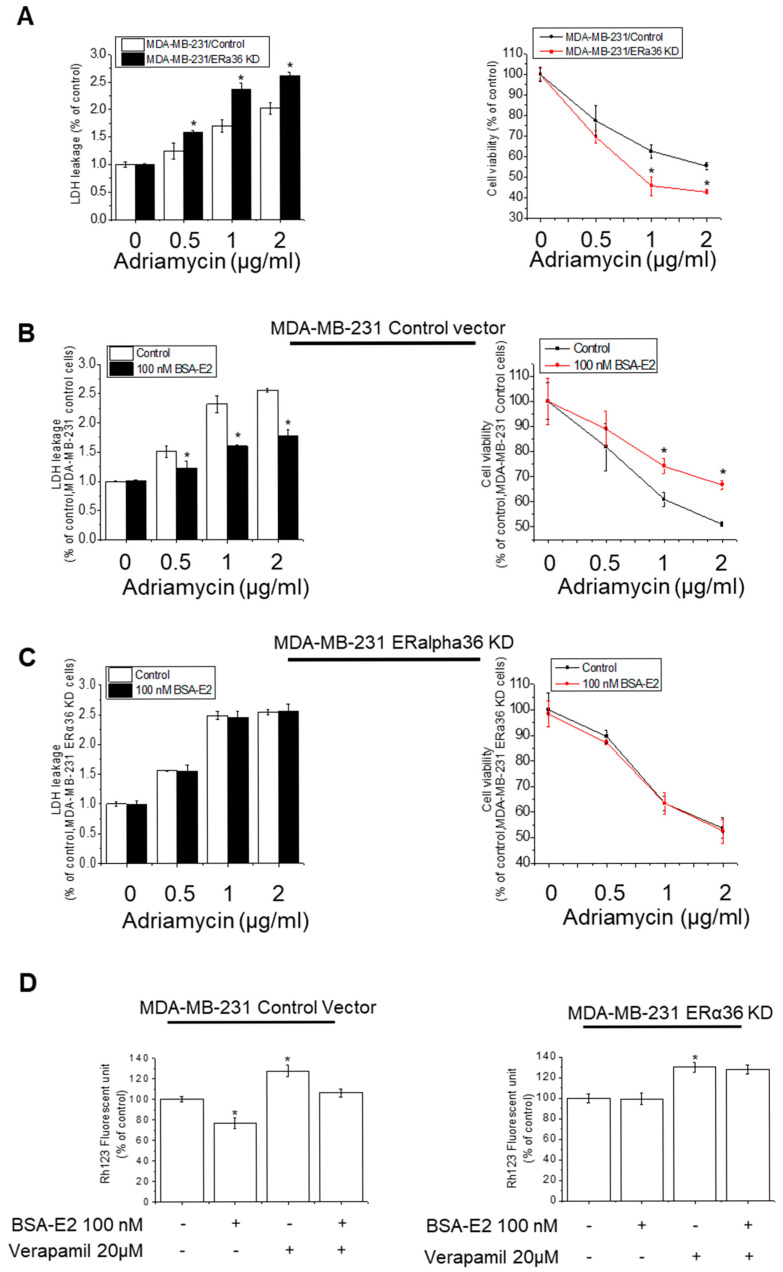
ERα36 is important for BSA-E2-induced drug resistance and cell survival. (**A**) Wild-type cells (MDA-MB-231/Control) and ERα36-knockdown cells (MDA-MB-231/ERα36KD) were treated with 0.5 to 2 µg/mL of Adriamycin for 48 h. The effect of ERα36 deletion on the cytotoxicity of Adriamycin in MDA-MB-231 cells was assessed by LDH assays and MTT assays. (**B**) Wild-type cells (MDA-MB-231/Control) and ERα36-knockdown cells (MDA-MB-231/ERα36KD) (**C**) were pretreated with 100 nM of BSA-E2 for 24 h, followed by incubation with 0.5 to 2 µg/mL of Adriamycin for 48 h. LDH and MTT assays were used to determine cytotoxicity. (**D**). Rh-123 assays with wild-type cells (MDA-MB-231/Control) and ERα36-knockdown cells (MDA-MB-231/ERα36KD). Data are expressed as mean ± SD of three independent experiments. * significantly different from control cells, *p* < 0.01.

Given the established role of membrane-associated signaling in BSA-E2-induced *MDR1* expression, we next investigated whether ERα36 contributes to this regulation by monitoring the cell viability and cytotoxicity in the ERα36 knocked down cells in response to Adriamycin. ERα36 knocked down cells (ERα36 KD) exhibited a notable decrease in cytotoxicity and viability compared with wild-type cells (Control) (Figure 6A). To further investigate the role of ERα36 in BSA-E2-induced drug resistance, we assessed the cell viability and cytotoxicity following treatment with 100 nM of BSA-E2. In ERα36-knockdown cells, BSA-E2 had no effect; however, in wild-type cells, BSA-E2 treatment led to a marked reduction in the cytotoxicity and a corresponding increase in cell viability (Figure 6B,C). Additionally, the downregulation of ERα36 influenced the expression of multidrug resistance genes and proteins, as indicated by Rh-123 accumulation. Treatment with BSA-E2 did not alter the intracellular level of Rh-123 in ERα36 KD cells, whereas a decrease was observed in wild-type cells, suggesting that ERα36 is required for BSA-E2-induced drug efflux activity, likely through the upregulation of multidrug resistance proteins. Collectively, these findings indicate that ERα36 contributes to drug resistance and cell survival in MDA-MB-231 cells.

### 2.7. ERα36 Is Important for Mediating BSA-E2-Induced MDR1 Expression Through Akt/ERK and NF-κB/CREB Signaling Pathway

**Figure 7:**
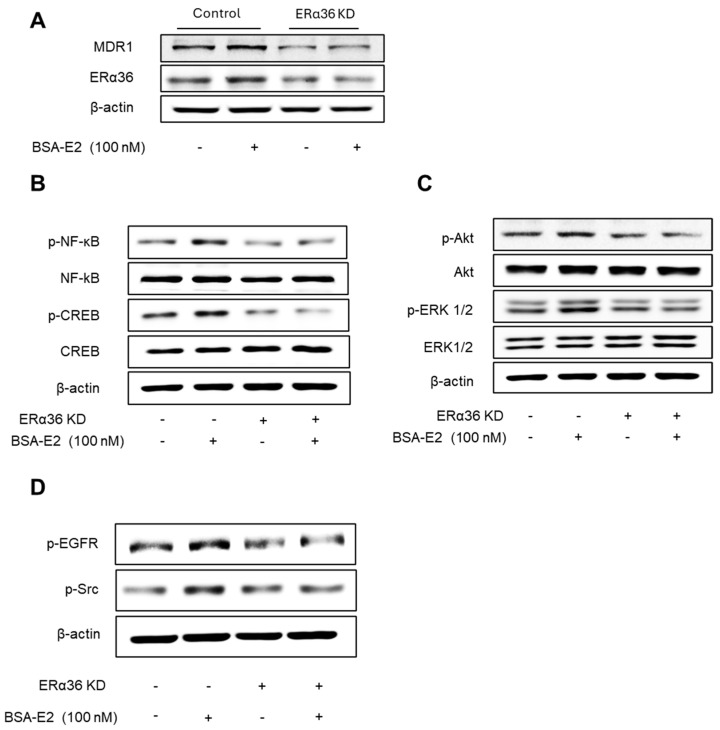
Effect of ERα36 on the protein expression of *MDR1* and the phosphorylation of NF-κB, CREB, Src, EGFR, Akt, and ERK. (**A**) Cells were treated with 100 nM of BSA-E2 for 24 h in control vector cells and ERα36 knockdown. *MDR1* and ERα36 protein levels were determined by Western blot. (**B**) Effect of ERα36 knockdown on NF-κB and CREB activation. Cells were treated with 100 nM of BSA-E2 for 15 min. p-NF-κB, NF-κB, p-CREB, and CREB protein levels were determined by Western blot. (**C**) Effect of ERα36 knockdown on the phosphorylation of Akt and ERK. Cells were treated with 100 nM of BSA-E2 for 15 min. p-AKT, Akt, p-ERK, and ERK protein levels were determined by Western blot. (**D**) Control and ERα36-knockdown cells were treated with 100 nM of BSA-E2 for 5 min, and p-EGFR and p-Src were detected by Western blot.

We next explored the role of ERα36 in regulating signaling pathways involved in BSA-E2-induced *MDR1* expression. ERα36 KD cells exhibited a markedly lower level of the MRD1 protein compared with wild-type cells (Figure 7A; quantified in Supplementary Figure S5A). Notably, BSA-E2 treatment failed to induce MRD1 expression in ERα36 KD cells, in contrast to the response observed in control cells. In addition, the ERα36 knockdown suppressed BSA-E2-induced phosphorylation of NF-κB, CREB, Akt, EGFR, and Src (Figure 7B–D; quantified in Supplementary Figure S5B–D). These results demonstrate that ERα36 is located on the plasma membrane and mediates BSA-E2-stimulated Akt/ERK activation through the Src/EGFR signaling pathway, resulting in increased *MDR1* expression.

### 2.8. ERα36 Mediates BSA-E2-Induced Activation of Wnt/β-Catenin Signaling and MDR1 Expression

**Figure 8:**
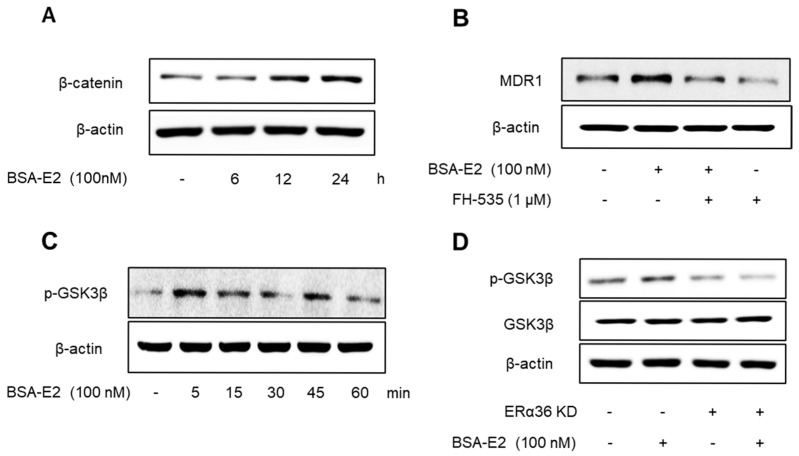
ERα36 is involved in BSA-E2-induced activation of the Wnt/β-catenin pathway to modulate *MDR1* expression. (**A**) Cells were pretreated with 100 nM of BSA-E2 for 6−24 h, and the β-catenin protein level was determined by Western blot. (**B**) *MDR1* expression was evaluated in cells pretreated with the Wnt/β-catenin inhibitor, FH-535 (1 µM) for 1 h and 100 nM of BSA-E2 for 24 h. (**C**) Cells were treated with 100 nM of BSA-E2 for 5−60 min, and the phosphorylation of GSK3β was determined by Western blot. (**D**) Control and ERα36-knockdown cells were treated with 100 nM of BSA-E2 for 24 h. p-GSK3 and GSK3β protein levels were analyzed by Western blot.

Emerging evidence suggests that the NF-κB/CREB signaling pathways interact functionally with the Wnt/β-catenin pathway to modulate gene expression. Therefore, we investigated whether ERα36 mediates its downstream effects through the activation of the Wnt/β-catenin signaling pathway. Treatment with BSA-E2 significantly increased the β-catenin levels (Figure 8A; quantified in Supplementary Figure S6A), while pretreatment with FH-535, a Wnt/β-catenin inhibitor, downregulated the expression of *MDR1* (Figure 8B and Figure S6B), suggesting that Wnt/β-catenin signaling plays a role in the regulation of *MDR1*. Notably, BSA-E2 treatment altered GSK3β phosphorylation, with a marked increase observed at 5 min post-treatment (Figure 8C and Figure S6C). This phosphorylation was abolished upon ERα36 knockdown (Figure 8D and Figure S6D). These data together reveal that the BSA-E2 activates the Wnt/β-catenin signaling pathway through an ERα36-dependent mechanism.

## 3. Discussion

P-glycoprotein, the product of the *MDR1* gene, has received great interest for its contribution to multidrug resistance in numerous cancer types [40]. The overexpression of *MDR1* is a major obstacle to effective cancer chemotherapy. Moreover, its role in enabling cancer cells to evade chemotherapy-induced apoptosis has been demonstrated in several cellular models, particularly in breast cancer cells, including TNBC [40,41]. TNBC characterized by a high expression of ERα36 has increased drug resistance, which leads to cell proliferation, metastasis, and malignancy, suggesting the potential significance of ERα36 status in predicting patient response to chemotherapy [25]. In this study, we provide evidence that ERα36 contributes to the acquisition of chemoresistance in TNBC by upregulating *MDR1* expression through a non-genomic pathway.

The activation of ERα36 by BSA-E2 significantly inhibited Adriamycin-induced cytotoxicity and markedly increased *MDR1* expression. Furthermore, treatment with BSA-E2 triggered the *MDR1*-dependent drug efflux evaluated by the reduced accumulation of its substrate, Rh-123. On the other hand, the silencing of ERα36 attenuated BSA-E2-induced *MDR1* expression and no longer exhibited effects on the suppression of Adriamycin-induced lethality. This suggests the contribution of ERα36 to estrogen-mediated drug resistance through the upregulation of *MDR1*. In the context of *MDR1* regulation, we explored the involvement of ERα36 in multiple interconnected signaling pathways.

### 3.1. ERα36-Akt/ERK/CREB and NF-κB Pathway

*MDR1* transcription is under the control of multiple transcription factors, such as NF-κB, CRE, AP-1, SP1, and PXR [42,43]. Our data demonstrate that BSA-E2 activates multiple non-genomic signaling pathways, notably the Akt/ERK/CREB and NF-κB axes. The inhibition of Akt or ERK suppressed BSA-E2-induced *MDR1* expression. Moreover, these effects were significantly attenuated upon the knockdown of ERα36, indicating that this receptor mediates the upstream activation of these kinases. The Akt and ERK pathways are well-established regulators of cell survival and drug resistance [19,44]. Furthermore, *MDR1* transcription is directly regulated by CREB and NF-κB through their binding to specific sites in the distal promoter region of the *MDR1* gene [35]. Phosphorylated CREB binds to its promoter region and enhances *MDR1* transcription. Similarly, NF-κB, especially the p65 (RelA) subunit, plays a critical role in *MDR1* regulation. Upon activation by cellular stress or chemotherapeutic agents, p65 translocates to the nucleus and binds to a consensus κB motif in the first intron of the *MDR1* gene, promoting its transcriptional activation [35,45]. This mechanism contributes to decreased intracellular drug accumulation and treatment failure in various cancer types. These results collectively support a model in which ERα36 activates the Akt/ERK signaling to drive *MDR1* regulation, contributing to enhanced drug resistance.

### 3.2. ERα36-EGFR/Src Signaling

We observed that BSA-E2 treatment rapidly induces phosphorylation of both EGFR and Src, whereas this signal is altered in ERα36 knockdown, suggesting a cross-correlation of action between EGFR and ERα36. ERα36 activates membrane-mediated estrogen signaling in association with EGFR and Src, resulting in the regulation of *MDR1* expression through the Akt and ERK signaling pathways. Indeed, a linkage between EGFR and ERα36 expression in carcinoma cells was determined, indicating that ERα36 influences the activation of extracellular signaling involved in EGFR/Src [16,44], probably through the interaction with membrane proteins of breast cancer cells. In particular, Src has been shown to serve as a key intermediary in this process by facilitating EGFR phosphorylation in response to estrogenic stimuli. Our data support this mechanism, implicating EGFR and Src as early effectors in the ERα36 signaling axis, ultimately contributing to *MDR1* regulation.

### 3.3. ERα36-Wnt/β-Catenin Pathway

Beyond classical kinase signaling, our data implicate the Wnt/β-catenin pathway as a downstream effector of ERα36 in promoting *MDR1* expression. The enhanced β-catenin protein levels and phosphorylation of GSK3β suggest that ERα36 plays roles in the activation of canonical Wnt signaling, which has been widely linked to tumor progression and multidrug resistance. It has been reported that the activation of the Wnt/β-catenin pathway enhances ABC transporter transcription, thereby contributing to chemoresistance across multiple cancer types [37,46]. In particular, the β-catenin/T-cell factor 4 transcriptional complex has been shown to directly target the *MDR1* gene and increase *MDR1* expression [47]. Furthermore, consistent with previous studies [48,49], our data demonstrated that the transactivation of *MDR1* can be inhibited by the pharmacological suppression of upstream regulators. Specifically, the inhibition of Src by PP2 and β-catenin by FH535 or the silencing of ERα36 effectively prevents *MDR1* expression, suggesting functional crosstalk between ER signaling and Wnt/β-catenin signaling pathways. Collectively, our data suggest that ERα36 simultaneously engages kinase cascades and developmental pathways like Wnt/β-catenin to orchestrate a coordinated response that enhances drug efflux and promotes resistance.

Taken together, our study reveals that ERα36 serves as a central hub in coordinating non-genomic estrogen signaling, promoting *MDR1* expression and resistance to Adriamycin. These effects are mediated through the activation of multiple pathways—Akt/ERK/CREB, NF-κB, and Wnt/β-catenin—as well as through interactions with EGFR. Importantly, the knockdown of ERα36 abolished most of these downstream events, confirming its essential role in this network. Our finding provides molecular evidence that a single receptor, like ERα36, can orchestrate diverse downstream oncogenic pathways. This highlights its potential as a therapeutic target to simultaneously overcome various mechanisms of drug resistance during treatment. Although our study focuses on breast cancer cells, particularly TNBC, the role of ERα36 may extend beyond this context. Previous studies have reported ERα36 expression in other cancer types, including lung, gastric, and endometrial cancers, suggesting its broader involvement in tumor biology. Future comparative studies are necessary to determine the generalizability of this mechanism. In addition, validation using animal models would be critical to investigate how ERα36 knockdown influences key tumor behaviors within the complex tumor microenvironments. Models such as patient-derived xenograft (PDX) or orthotopic mouse models could offer physiologically relevant settings that closely mimic patient responses to chemotherapy [50]. Furthermore, innovative drug delivery platforms, such as nanoparticle-based therapeutics, represent promising strategies to effectively target ERα36 in resistant cancer types [51].

These findings not only elucidate a new mechanism of estrogen-induced multidrug resistance in breast cancer but also point to ERα36 as a potential therapeutic target, particularly in tumors that are ERα-negative but still responsive to estrogen at the membrane level. Future studies may focus on identifying specific inhibitors of ERα36 or targeting its associated pathways to overcome resistance and improve chemotherapeutic efficacy.

## 4. Materials and Methods

### 4.1. Chemicals and Reagents

Dulbecco’s modified Eagle’s medium (DMEM), fetal bovine serum (FBS), penicillin–streptomycin, and trypsin were purchased from Welgene (Gyeongsan, South Korea). Antibodies against ERα36 were purchased from Alpha Diagnostic International Inc. (San Antonio, TX, USA). BSA-conjugated estradiol (BSA-E2) was obtained from Sigma-Aldrich (St. Louis, MO, USA). The following primary antibodies from Cell Signaling Technology (Danvers, MA, USA) were used at a 1:1000 dilution: *MDR1* (Cat#13342S), p-EGFR (Tyr1068 specific, Cat#2234S), EGFR (Cat#2232L), p-c-Src (Tyr416 specific, Cat#2101S), p-Akt (Ser472 specific, Cat#9271), Akt (Cat#9272S), p-ERK (Thr202/Tyr204 specific, Cat#9101), ERK (Cat#9102), p-NF-κB p65 (Ser536 specific, Cat#3033)), NF-κB p65 (Cat#8242), CREB (Cat#4820), p-CREB (Ser133 specific, Cat#9198). HRP-conjugated anti-rabbit (Cat#sc-2357) or anti-mouse (Cat#sc-2005) secondary antibodies from Santa Cruz Biotechnology (Dallas, TX, USA) were used at a 1:5000 dilution. Verapamil, rhodamine, and Adriamycin were purchased from the Sigma Chemical Company (St. Louis, MO, USA). RNAiso was purchased from Takara Bio (Shiga, Japan). Antibody against β-actin was purchased from Santa Cruz Biotechnology (Santa Cruz, CA, USA). 3-(4,5-Dimethylthiazol-2-yl)-2,5-diphenyltetrazolium bromide, a tetrazole) (MTT) was purchased from USB Corporation (Cleveland, OH). All other commercially available chemicals used were of the highest purity.

### 4.2. Cell Culture and Cell Viability Assays

The human breast cancer cell line MDA-MB-231 was provided by the American Type Culture Collection (ATCC, Manassas, VA, USA). Cells were grown in DMEM supplemented with 10% FBS, 2 mM L-glutamine, 100 U/mL of penicillin, and 100 μg/mL streptomycin at 37 °C in an atmosphere containing 5% CO_2_. To assess cell viability, cells were plated in 48-well plates at 2 × 10^4^ cells/well, and 100 nM of BSA-E2 was added to each well after 24 h incubation. The MTT and LDH assays were performed as described previously [52]. Briefly, cells were treated with MTT for 1 h, and formazan crystals were solubilized with DMSO. Absorbance was measured at 570 nm with a Biotek Synergy HT microplate reader (BioTek Instruments, Winooski, VT, USA). LDH activity in the supernatant was determined at 490 nm.

### 4.3. Lentiviral Vector Production and Transduction

Lentiviral ERα36 shRNA vectors (HSH859L-1-LVRU6GP) for ERα36 knockdown and lentiviral control (CSHCTR00-LVRU6GP) vectors were purchased from Genecopoeia (Rockville, MD, USA). Following the manufacturer’s instructions, all lentiviral titers were produced using a Lenti-PacTM FIV Expression Packaging Kit (Genecopoeia, MD, USA). ERα36-knockdown stable cell lines were generated by transducing MDA-MB-231 cells with purified virus, followed by a selection of stable pools of cells using 5 μg/mL of puromycin (Sigma-Aldrich, St. Louis, MO, USA).

### 4.4. RNA Extraction and qRT-PCR

Total RNA was extracted from harvested cell pellets containing approximately 1 × 10^6^ MDA-MB-231 cells using RNAiso Plus (total RNA extraction reagent; Takara Bio, Shiga, Japan). After RNA isolation, cDNA was synthesized using the BioFact RT Series kit (BioFact, Daejeon, Korea). The qRT-PCR results were analyzed using Bio-Rad CFX Connect Real-Time PCR software, version 1.4.1 (Bio-Rad Laboratories, Hercules, CA, USA). The PCR primers were used as follows. The expression was normalized with the endogenous control, glyceraldehyde 3-phosphate dehydrogenase (GAPDH). *MDR1* (NM_001348945.2) forward: 5′-GCTGTCAAGGAAGCCAATGCCT-3′, *MDR1* (NM_001348945.2) reverse: 5′-TGCAATGGCGATCCTCTGCTTC-3′; GAPDH (NM_001256799.3) forward: 5′-GTCTCCTCTGACTTCAACAGCG-3′, GAPDH (NM_001256799.3) reverse: 5′-ACCACCCTGTTGCTGTAGCCAA-3′.

### 4.5. Western Blotting

Western blotting was performed according to the standard protocol [31]. Briefly, approximately 1 × 10^7^ cells were harvested and lysed using CETi lysis buffer (TransLab, Daejeon, Korea). An equal amount of total protein was separated by sodium dodecyl sulfate polyacrylamide gel electrophoresis (SDS-PAGE) and transferred onto a nitrocellulose membrane. The membranes were blocked using skim milk for 1 h. Subsequently, primary antibodies were incubated at 4 °C overnight, and secondary antibodies were incubated at RT for 2 h. The membrane was exposed using the Hisol ECL Plus detection kit (Biofact, Daejeon, Korea).

### 4.6. Rhodamine-123 Accumulation Assay

Cells were plated onto 24-well plates (at 10^5^ cells/well) and pretreated with 10−100 nM of BSA-E2 and 20 μM of verapamil for 48 hr. Verapamil was used as a positive control for MDR inhibition [53]. Following pretreatment, cells were cultured in medium containing 5 μM of Rh-123 for 90 min, protected from light. Cells were trypsinized, washed twice with ice-cold PBS, and resuspended in 1 mL of PBS. Intracellular Rh123 accumulation was measured by fluorescence at 488 nm excitation and 530 nm emission using a BioTek Synergy HT microplate reader (BioTek Instruments, Winooski, VT, USA).

### 4.7. Statistical Analysis

All experiments were performed in triplicate. The data are reported as mean ± SD of independent experiments. The Shapiro–Wilk test was used for data normality. The statistical evaluation of the results was performed using one-way ANOVA. The Tukey–Kramer test was used for multi-group comparisons. Statistical significance was defined as *p* < 0.01.

## 5. Conclusions

In summary, this study demonstrates that ERα36 contributes to drug resistance in TNBC cells by promoting *MDR1* expression through multiple non-genomic pathways, including Akt/ERK, NF-κB, CREB, and Wnt/β-catenin. These findings expand the functional repertoire of ERα36 beyond tumor growth and suggest its involvement in regulating key resistance mechanisms, positioning it as a potential therapeutic target in drug-resistant breast cancers.

## Data Availability

Data is contained within the article.

## Supplementary Materials

The following supporting information can be downloaded at: https://www.mdpi.com/article/10.3390/ijms26157200/s1.

## Abbreviations

The following abbreviations are used in this manuscript:

Akt	Protein kinase B
BSA-E2	BSA-conjugated estradiol
CREB	cAMP-responsive element binding protein
CBP	CREB binding protein
EGFR	Epidermal growth factor receptor
EMT	Epithelial–mesenchymal transition
ERs	Estrogen receptors
Erα66	Estrogen receptor 66 alpha
Erα36	Estrogen receptor 36 alpha
ERK	Extracellular Signal-Regulated kinase
HER2	Human epidermal growth factor receptor 2
MAPK	Mitogen-activated protein kinase
*MDR1*	Multidrug resistance 1
NF-κB	Nuclear factor-κB
P-glycoprotein	P-gp
PR	Progesterone receptors
PI3K	Phosphatidylinositide3-kinase
Rh-123	Rhodamine 123
Src	Proto-oncogene tyrosine protein kinase
TNBC	Triple-negative breast cancer
WBP2	WW domain-binding protein 2
