# Supplementary material for: ERα36 Promotes MDR1-Mediated Adriamycin Resistance via Non-Genomic Signaling in Triple-Negative Breast Cancer
Source: Int J Mol Sci. 2025 Jul 25;26(15):7200. doi: 10.3390/ijms26157200 (PMC12346883; doi:10.3390/ijms26157200)
Supplement: Supplementary file 1 [file ijms-26-07200-s001.zip › ijms-3766575-supplementary.pdf]

ERα36 promotes MDR1-mediated Adriamycin resistance via non-genomic signaling in triple-negative breast cancer

Muslimbek Mukhammad Ugli Poyonov 1, †, Anh Thi Ngoc Bui 1, †, Seung Yeon Lee 1, †, Gi Ho Lee 1 and Hye Gwang Jeong 1,*

[1] College of Pharmacy, Chungnam National University, Daejeon 34134, Republic of Korea
* Correspondence: hgjeong@cnu.ac.kr; Tel.: +82-42-821-5936; Fax: +82-42-823-6566.
† These authors contributed equally to this work.

Figure S1.

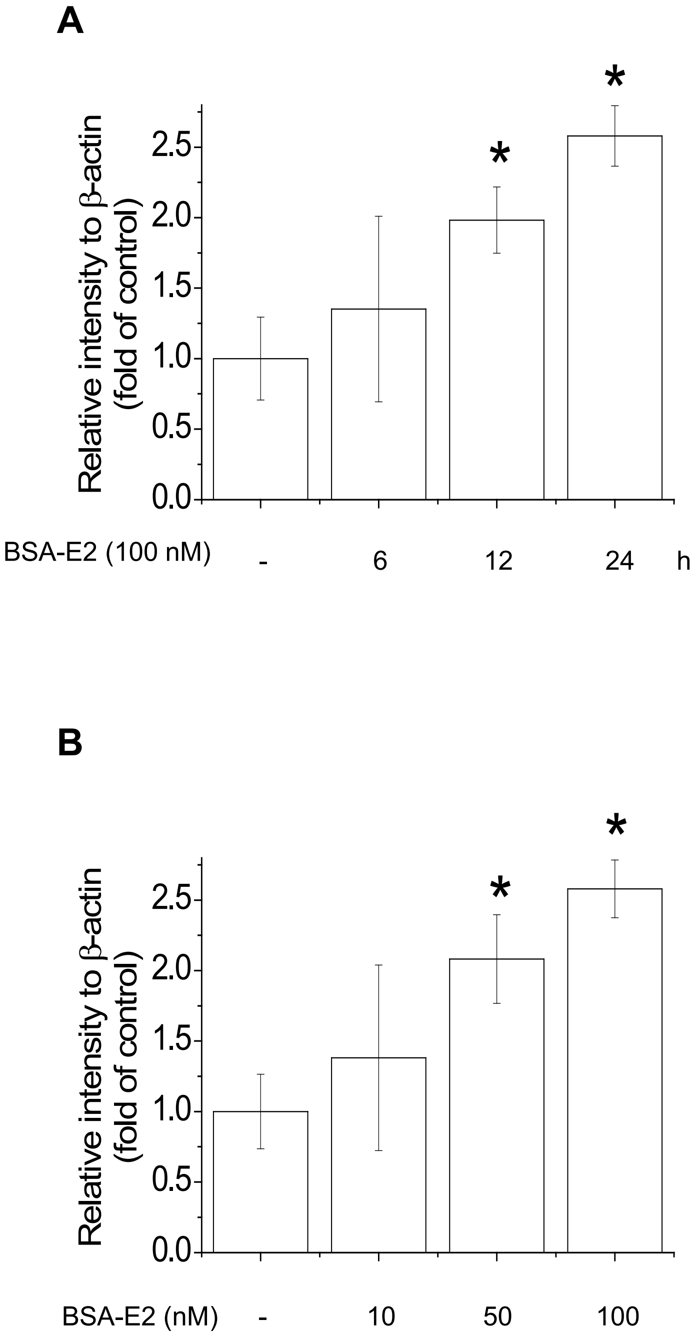

Figure S1. BSA-E2 induces MDR1 expression in MDA-MB-231 cells. (A) Quantification of band intensity from Figure 2C, normalized to the loading control. (B) Quantification of band intensity from Figure 2D, normalized to the corresponding loading control. The band intensity was analyzed using the ImageJ program. All experiments were performed thrice (n=3). Data are expressed as the mean ± SD. *p< 0.01 compared to the control group.

Figure S2.

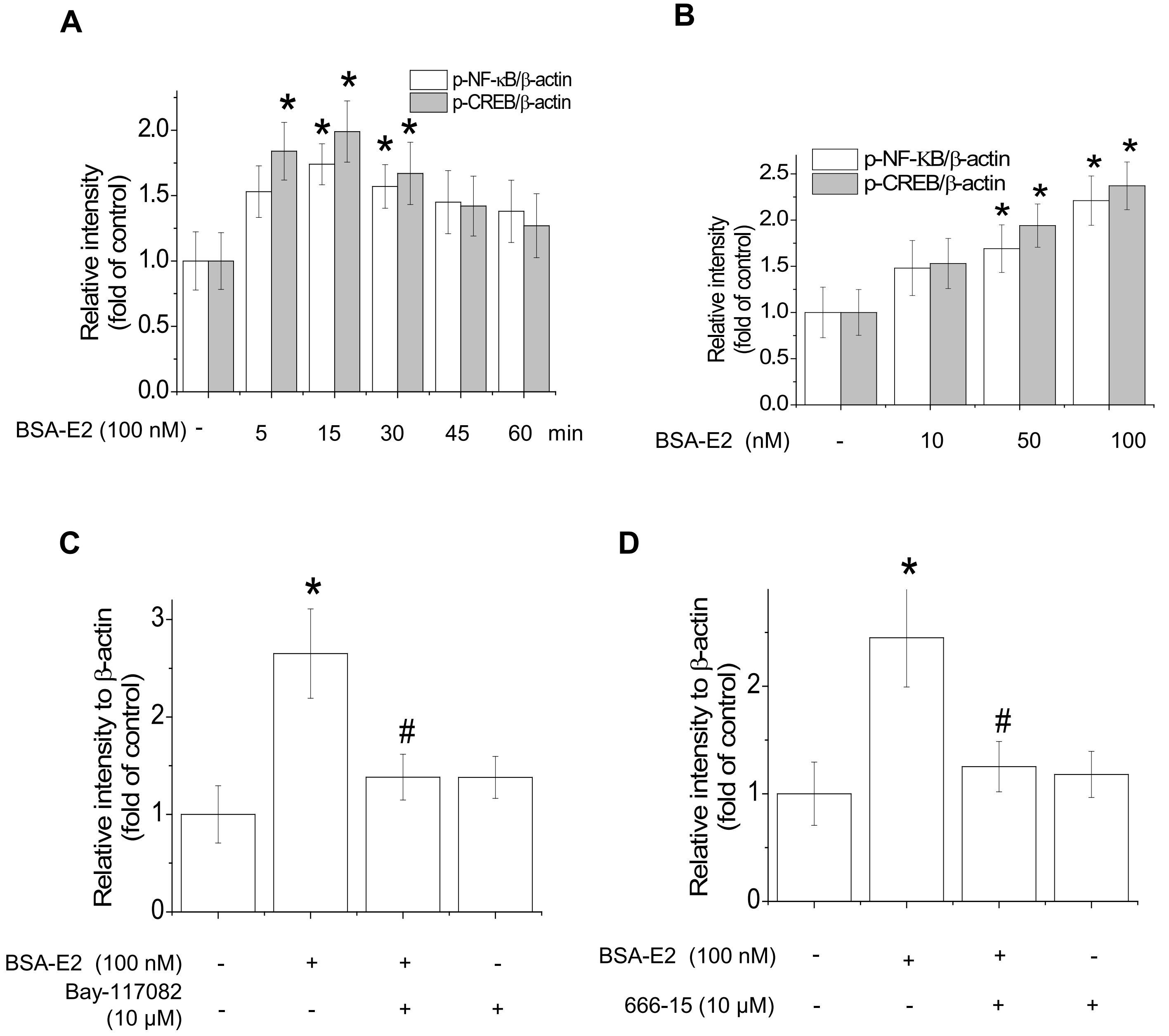

Figure S2. BSA-E2 enhances MDR1 level via activation of NF-κB and CREB phosphorylation. (A) Quantification of band intensity from Figure 3A, normalized to the loading control. (B) Quantification of band intensity from Figure 3B, normalized to the corresponding loading control. (C and D) Quantification of band intensity from Figures 3C and 3D, normalized to the corresponding loading control. The band intensity was analyzed using the ImageJ program. All experiments were performed thrice (n=3). Data are expressed as the mean ± SD. *p< 0.01 compared to the control group. #p< 0.01 compared with the BSA-E2 treatment group.

Figure S3.

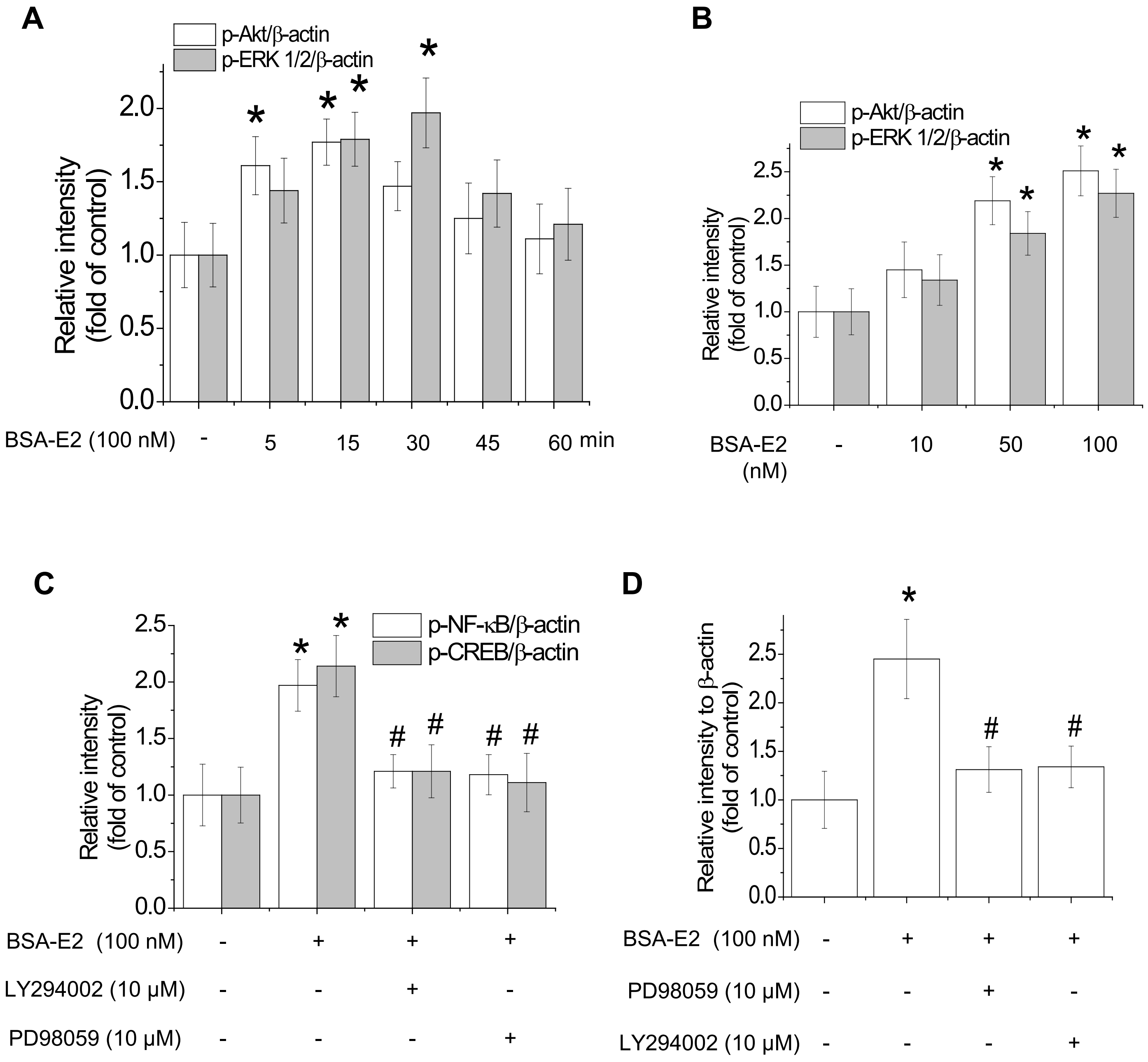

Figure S3. The Akt/ERK signaling pathway is important for the ERα36-mediated regulation of MDR1 expression. (A and B) Quantification of band intensity from Figure 4A and 4B, normalized to the loading control. (C and D) Quantification of band intensity from Figures 4C and 4D, normalized to the corresponding loading control. The band intensity was analyzed using the ImageJ program. All experiments were performed thrice (n=3). Data are expressed as the mean ± SD. *$p < 0.01$ compared to the control group. #$p < 0.01$ compared with the BSA-E2 treatment group.

Figure S4.

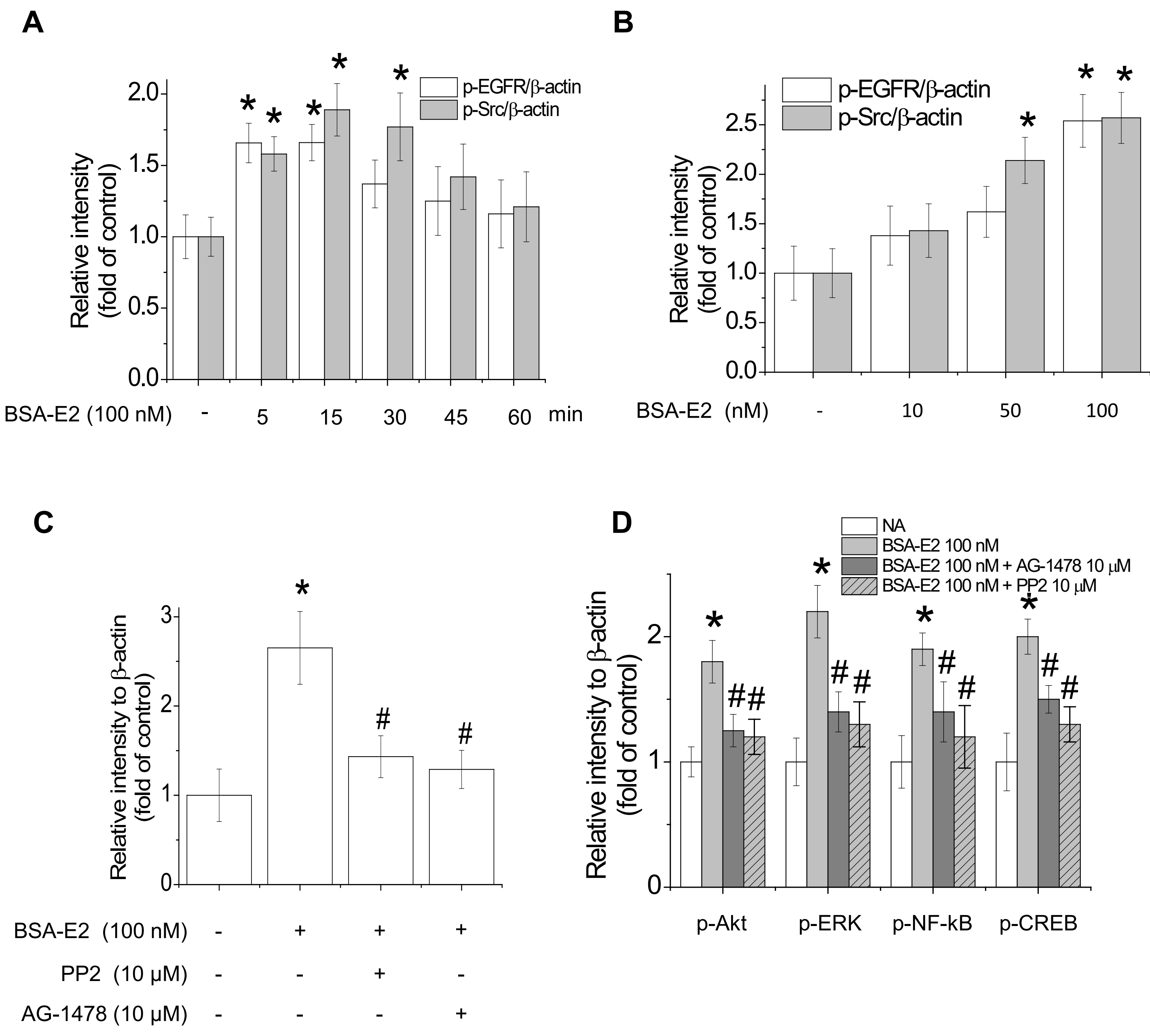

Figure S4. Effect of BSA-E2 on MDR1 expression with the Src/EGFR signaling pathway. (A and B) Quantification of band intensity from Figure 5A and 5B, normalized to the loading control. (C and D) Quantification of band intensity from Figures 5C and 5D, normalized to the corresponding loading control. The band intensity was analyzed using the ImageJ program. All experiments were performed thrice (n=3). Data are expressed as the mean ± SD. *p< 0.01 compared to the control group. #p< 0.01 compared with the BSA-E2 treatment group.

Figure S5.

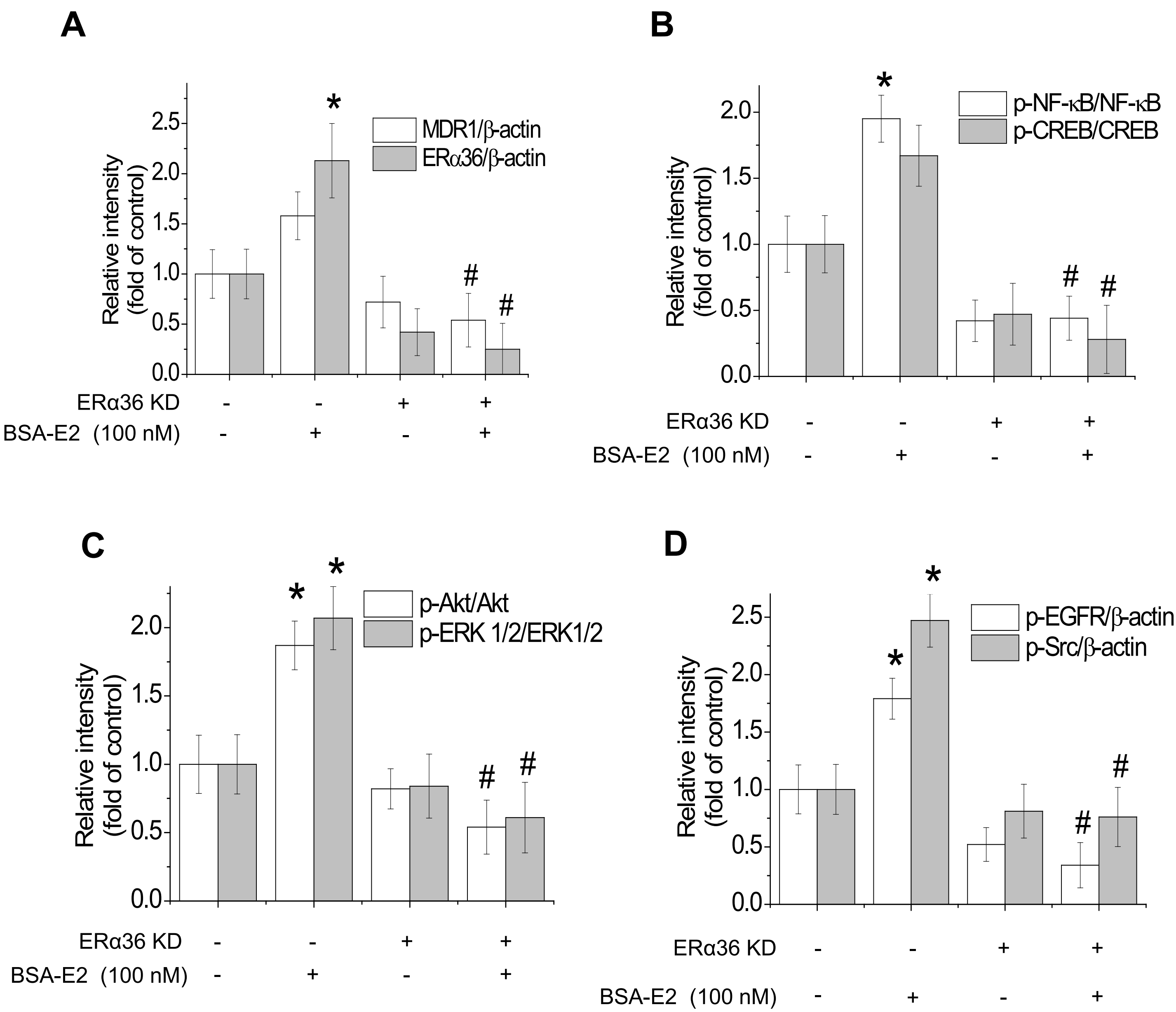

Figure S5. Effect of ERα36 on the protein expression of MDR1 and phosphorylation of NF-κB, CREB, Src, EGFR, Akt, and ERK. (A and B) Quantification of band intensity from Figure 7A and 7B, normalized to the loading control. (C and D) Quantification of band intensity from Figures 7C and 7D, normalized to the corresponding loading control. The band intensity was analyzed using the ImageJ program. All experiments were performed thrice (n=3). Data are expressed as the mean ± SD. *$p < 0.01$ compared to the control group. #$p < 0.01$ compared with the BSA-E2 treatment group.

Figure S6.

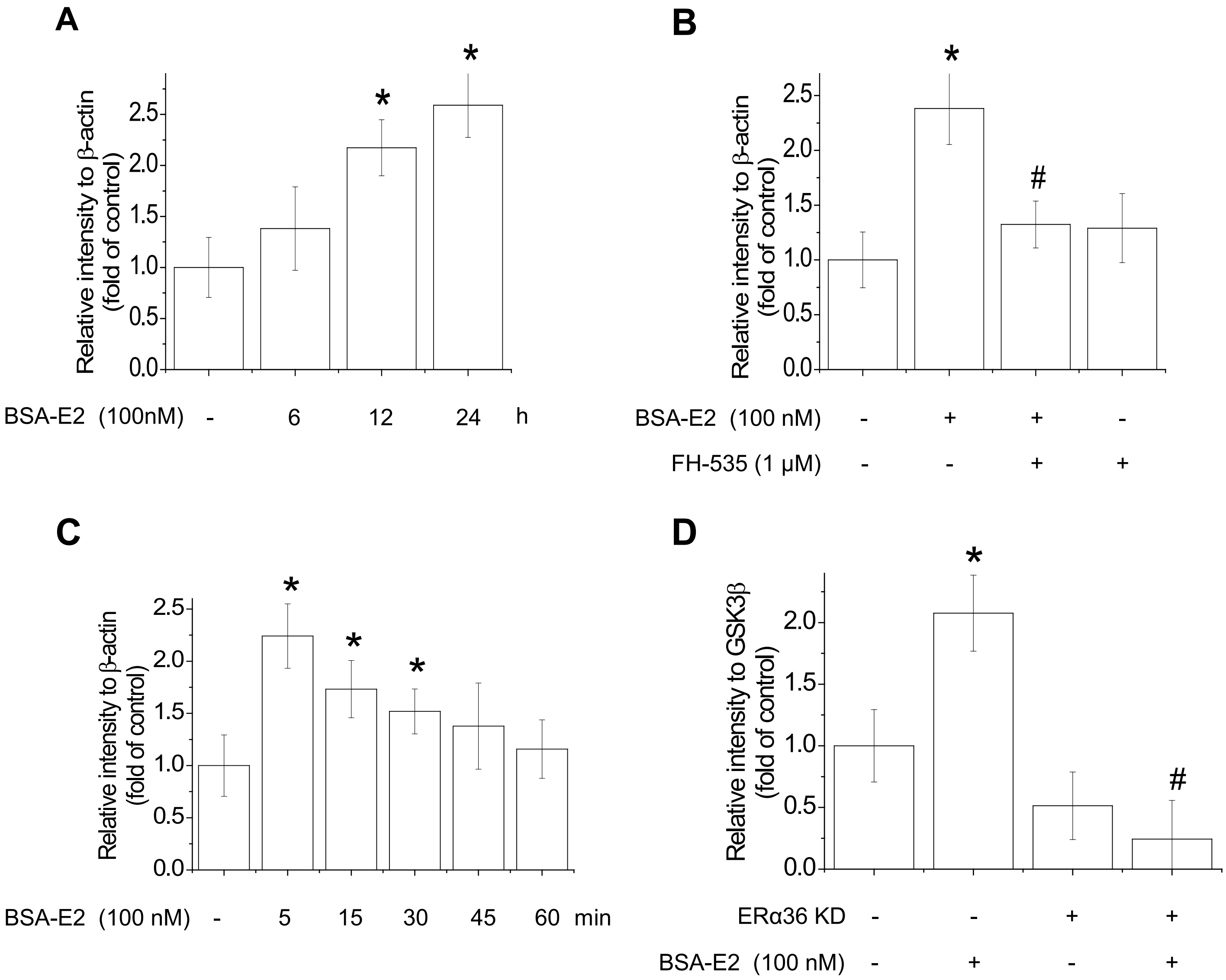

Figure S6. ERα36 is involved in BSA-E2-induced activation of the Wnt/β-catenin pathway to modulate MDR1 expression. (A and B) Quantification of band intensity from Figure 8A and 8B, normalized to the loading control. (C and D) Quantification of band intensity from Figures 8C and 8D, normalized to the corresponding loading control. The band intensity was analyzed using the ImageJ program. All experiments were performed thrice (n=3). Data are expressed as the mean ± SD. *p< 0.01 compared to the control group. #p< 0.01 compared with the BSA-E2 treatment group.
